# Flower-Based Green Synthesis of Metallic Nanoparticles: Applications beyond Fragrance

**DOI:** 10.3390/nano10040766

**Published:** 2020-04-16

**Authors:** Harsh Kumar, Kanchan Bhardwaj, Kamil Kuča, Anu Kalia, Eugenie Nepovimova, Rachna Verma, Dinesh Kumar

**Affiliations:** 1 School of Bioengineering & Food Technology, Shoolini University of Biotechnology and Management Sciences, Solan-173229, H. P., India; microharshs@gmail.com; 2 School of Biological and Environmental Sciences, Shoolini University of Biotechnology and Management Sciences, Solan-173229, H. P., India; kanchankannu1992@gmail.com (K.B.); rachnaverma@shooliniuniversity.com (R.V.); 3 Department of Chemistry, Faculty of Science, University of Hradec Kralove, Hradec Kralove 50003, Czech Republic; eugenie.nepovimova@uhk.cz; 4 Electron Microscopy and Nanoscience Laboratory, Punjab Agricultural University, Ludhiana-141004, Punjab, India; kaliaanu@pau.edu

**Keywords:** flower extract, green synthesis, nanoparticles, phytochemicals, antibacterial, antioxidants, catalytic, insecticidal

## Abstract

Green synthesis has gained wide attention as a sustainable, reliable, and eco-friendly approach to the synthesis of a variety of nanomaterials, including hybrid materials, metal/metal oxide nanoparticles, and bioinspired materials. Plant flowers contain diverse secondary compounds, including pigments, volatile substances contributing to fragrance, and other phenolics that have a profound ethnobotanical relevance, particularly in relation to the curing of diseases by ‘Pushpa Ayurveda’ or floral therapy. These compounds can be utilized as potent reducing agents for the synthesis of a variety of metal/metal oxide nanoparticles (NPs), such as gold, silver, copper, zinc, iron, and cadmium. Phytochemicals from flowers can act both as reducing and stabilizing agents, besides having a role as precursor molecules for the formation of NPs. Furthermore, the synthesis is mostly performed at ambient room temperatures and is eco-friendly, as no toxic derivatives are formed. The NPs obtained exhibit unique and diverse properties, which can be harnessed for a variety of applications in different fields. This review reports the use of a variety of flower extracts for the green synthesis of several types of metallic nanoparticles and their applications. This review shows that flower extract was mainly used to design gold and silver nanoparticles, while other metals and metal oxides were less explored in relation to this synthesis. Flower-derived silver nanoparticles show good antibacterial, antioxidant, and insecticidal activities and can be used in different applications.

## 1. Introduction

The theoretical concept of nanotechnology was first described in 1959 by the physicist, Richard Feynman [1]. Nanotechnology is defined as understanding, controlling, and manipulating matter at the level of individual atoms and molecules [2]. Metal nanoparticles (NPs) with distinct physico-chemical properties have gained considerable attention in the last few decades [3]. Due to their ultra-small size and large surface area to volume ratio, a great interest in the use of NPs—which display variations in both physical and chemical properties, as compared to the bulk of similar chemical compositions—has developed [4,5,6]. As a result of their unique optoelectronic and physico-chemical properties, NPs have a number of applications, including their use as catalysts, electronic components, and chemical sensors in medical diagnostic imaging, medical treatment protocols, and pharmaceutical products [7]. 

Nanoparticles can be synthesized using two different fundamental approaches (top down and bottom up methods) to obtain nanomaterials with a desired shape, size, and functionality [8]. The former involves the generation of nanomaterials/nanoparticles using diverse synthesis approaches, like ball milling, lithographic techniques, etching, and sputtering [9]. The bottom-up approach usually used to synthesize nanoparticles normally involves aggressive reducing agents (hydrazine and sodium borohydride), along with a capping agent and volatile solvent, like chloroform and toluene. These methods are effective in synthesizing well-defined and pure metallic nanoparticles, but their production cost remains the main hinderance [10]. Therefore, there is a need for the development of a cost-effective and environmentally friendly alternative, which would allow an eco-friendly reducing agent, environmentally compatible solvents and nonhazardous capping agents to be used for the synthesis of nanoparticles. All these criteria have been proposed as the primary prerequisite for green nanoparticle synthesis [11].This review focuses on the use of flower extracts for the green synthesis of several types of nanoparticles and their applications. It also highlights the key challenges of green flower-mediated nanoparticles.

## 2. Importance of Flowers in Daily Life

There is a special association between humans and flowers, and the aesthetic appeal of flowers triggered humans to cultivate flowers and propagate them, just like insects do with pollen [12,13]. Flowers have an attractive visual quality, and vision is a multimodal procedure that activates visual regions of the brain, as well as the viscera-motor, sensory-motor and affective cerebral circuits. Various parts of the brain are activated by flowers, creating an interesting perceptual experience [14]. Flowers also induce a multisensory experience, as observed while watching flowers sway in the wind and their use in perfume [15,16]. Additionally, in the Ayurveda and Siddha systems, some flowers have been reported to possess distinct medicinal properties [17]. In rasayana medicines, about 18,000 kinds of flowers have been mentioned [18]. An ayurvedic text,“Kaiyadevanighantu”, mainly describes the flowers of many medicinal plants as having therapeutic benefits [19]. 

Due to the vast and ancient knowledge of health care, the contemporary medical challenges can possibly be tackled through research on the phytochemical constituents present in flowers and their pharmacological properties. The phytochemical analysis of the *Hibiscus rosa-sinensis* flower shows the presence of constituents, such as indole alkaloids, saponins, reducing sugars, tannins, and terpenoids;while their aqueous extracts may contain cardiac glycosides and flavonoids, such as cyanidin, quercetin, and saponins [20,21]. Most of these secondary metabolites are responsible for antibacterial activities or possess haemo-protective properties [22,23,24,25]. The flower of *Mimusops elengi* contains 74 different compounds belonging to flavonoids, alkaloids, phenolics, and tannins, which can be isolated using various extraction methods [26,27,28]. Methanolic extract has been reported to inhibit the growth of a number of bacterial pathogens [29,30]. An anti-malarial compound, cyclohexyl ethanoid (rengyolone), isolated from the ethanolic extract of the *Nyctanthes arbor-tristis* flower, has been reported to be effective against *Plasmodium falciparum* [31]. Another compound, benzofuranone, 3, 3a, 7, 7a-tetrahydro-3a hydroxy-6(2H)-benzofuranone, was also isolated from this flower and exhibits a significant antibacterial activity against both Gram-negative and Gram-positive bacteria [32]. Furthermore, there are also reports indicating that the antidiabetic activity of the *Nyctanthes arbor-tristis* flower extract is more effective than the leaf extract [33]. *Tussilago farfara* flower buds yield two flavonoids, namely, quercetin 3-O-beta-D-glucopyranoside and quercetin 3-O-beta-L-arabinopyranoside, with a higher antioxidative activity than their aglycone and quercetin, as shown by a nitro blue tetrazolium (NBT) superoxide scavenging assay [34]. The diverse compounds present in various flower extracts can act as oxidizing/reducing agents or as biotemplates to aid in the green synthesis of NPs, particularly metal/metal oxide NPs.

## 3. Green Synthesis of Nanoparticles (NPs)

**Figure 1:**
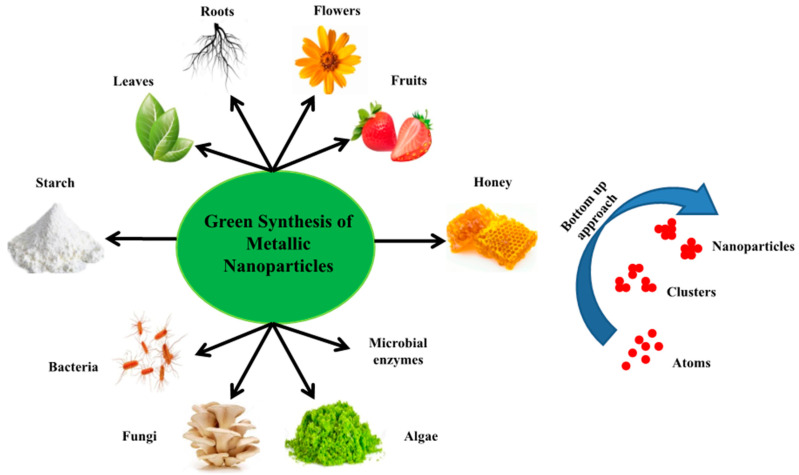
Different types of green synthesis used for the preparation of metal nanoparticles.

Green-synthesized NPs can be obtained through an easy, efficient, economical and eco-friendly biological synthesis approach [35]. Metallic nanoparticles can be obtained from cell or cell-free extracts of a variety of biological resources, as shown in Figure 1. The key factor that should be considered during the nanoparticle preparation is that it should be evaluated against green chemistry principles, like the selection of a solvent medium, eco-friendly reducing agent, and non-toxic material for nanoparticle stabilization [36]. Furthermore, compounds like peptides, polyphenolics, sugars, vitamins, and water from coffee and tea extracts were found to be appropriate for the synthesis of nanoparticles [37,38,39,40,41,42]. As compared to microbial NPs, plant-based NPs are more stable and monodispersed, and plant extract takes less time to reduce metal ions. Microbial synthesis is one of the approaches to the synthesis of nanomaterials. 

Prokaryotic bacterial cell/cell extracts have been reported in relation to the synthesis of a variety of NPs, including cadmium sulfide (CdS), gold (Au), silver (Ag), silver oxide (AgO), and titanium dioxide (TiO_2_) [43,44,45,46,47,48,49]. Some fungi have also been used for the synthesis of CdS, Ag, and TiO_2_ NPs [45,47,50,51,52,53]. Recently, gold, iron oxide, silver, and zinc oxide NPs have been synthesized using algae [54,55,56,57,58,59]. Likewise, leaf, seed, and root extracts, latex and bulbs of plants have also been utilized for the synthesis of Ag, palladium (Pd), and Au NPs [60,61,62,63,64,65,66,67,68,69]. Other materials of a biological origin, such as honey, can also synthesize carbon, Ag, Au, Pd, and platinum (Pt) nanoparticles [70,71,72,73,74]. 

## 4. Green Synthesis of Nanoparticles Mediated by Flowers

**Figure 2:**
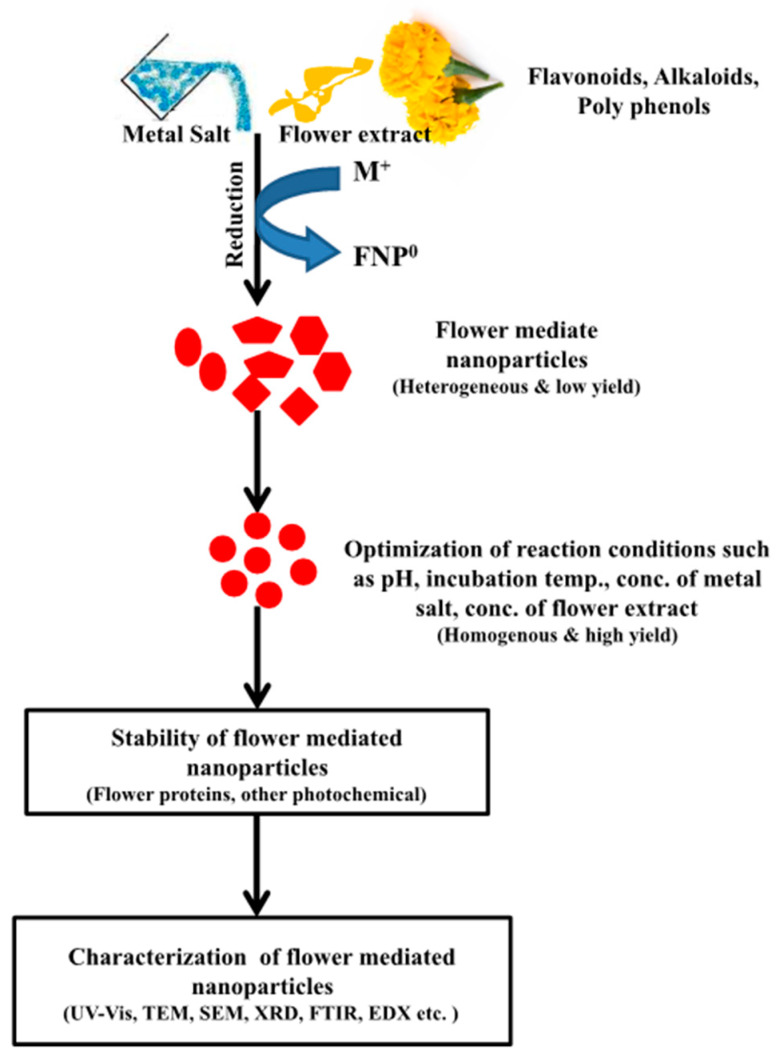
A general mechanism of nanoparticle formation from flower extract. M^+^ (metal); FNP^0^ (flower nanoparticles).

Flowers have unique chemical properties that can be useful for nanoparticle synthesis. The synthesis of flower-mediated NPs is advantageous, as compared with other biological NPs synthesis methods, particularly the one mediated through microorganisms, as microorganisms need to be maintained or cultured under aseptic and pure culture conditions. It is a difficult task to separate nanoparticles during the downstream processing of microbial broth cultures. Furthermore, it takes more time to convert soluble metallic salts to elemental or element oxide NPs. A generalized mechanism (Figure 2) for the biosynthesis of different nanoparticles using flower extracts has been summarized in Table 1. The various types of nanoparticles derived from different flower extracts are discussed in the following sections.

### 4.1. Silver Nanoparticles (AgNPs)

Silver nanoparticles (AgNPs) show a considerably large surface area, which leads to a significant biochemical reactivity, catalytic action, and atomic behavior, when compared with large particles with an identical chemical configuration [88]. The synthesis of noble AgNPs is a two-step procedure that first involves the reduction of Ag^+^ ions to Ag^0^, and after this agglomeration and stabilization is completed, the synthesis involves the development of oligomeric clusters of colloidal AgNPs [89]. The reduction procedure occurs in the presence of biological catalysts. The flower-derived AgNPs have shown numerous applications, which are given in Table 2. 

### 4.2. Gold Nanoparticles (AuNPs)

Extensive attention has been paid to gold nanoparticles (AuNPs) due to their good shape, size, optical characteristics, and biocompatibility [98]. AuNPs of several sizes and morphologies have gained significant attention in relation to applications in the field of medicine, i.e., as carriers for drugs, such as paclitaxel, tumor-detectors, photothermal agents, or radiotherapy dose enhancers [99,100,101,102,103]. Flower-mediated AuNPs have also shown antimicrobial and catalytic activity, which is shown in Table 3.

### 4.3. Other Nanoparticles

Metal nanoparticles based on titanium (Ti), cadmium (Cd), copper (Cu), iron (Fe), zinc (Zn), and magnesium (Mg), etc., have been emerging as a new class, owing to their exclusive applications in research (Table 4). Rosemary extract (*Rosmarinus officinalis* L.) was used in MgO nano-flower synthesis in a stirring situation at 70 °C for 4 h [86]. Marigold flower (*Tageta sp.*) petal extract was used in the synthesis of cadmium nanoparticles (CdNPs) [84]. In this study, a solution of cadmium chloride (88 ml) and petal extract (12 ml) was mixed, which resulted in a yellow nanoparticle solution with a sphereshape, as observed under a fluorescent microscope. In the combustion method, zinc nitrate [Zn(NO_3_)_2_·6H_2_O] was used as a substrate to synthesize ZnO NPs using *Syzygium aromaticum* bud and flower extract. The solution was poured into a China dish and stirred for 5–10 min at a constant temperature of 400± 10°C in a muffle furnace for 4 min to complete the entire combustion process. The synthesized SaZnO NPs of an off-white color were obtained as the final product [105]. An aqueous flower extract of *Piliostigma thonningii* was also used in the synthesis of iron nanoparticles by reacting the flower extract with a ferrous chloride solution. Reductants already present in the flower extract functioned both as reducing and stabilizing agents [87]. A *Calotropis gigantean* flower extract was used for the synthesis of titanium dioxide nanoparticles (TiO_2_ NPs) [85]. *Mimusops elengi* flower powder was used to synthesize CuNPs and showed good antibacterial, anti-coagulant, antifungal, and anti-larval activities [106]. 

## 5. Approaches Used in the Characterization of Nanoparticles

Metallic nanoparticles synthesized from extracts of several flowers of a diverse size, shape, and surface areas are categorized using different approaches, as shown in Table 5. The composition, size, structure, and crystal phase of the synthesized nanoparticles are deduced using UV–vis, XRD, FT-IR, DLS, EDS, and Raman spectroscopy. The range of the UV spectra wavelength, from 300 to 800 nm, illustrates the existence of several metallic nanoparticles of a size ranging from 2 nm to 100 nm. Usually, the detection of gold nanoparticles is conducted using UV spectroscopy in the range of 500 and 580 nm [108]. Estimation of the size of the synthesized nanoparticles, along with the quantification of the charges on the surface of the nanoparticles, is conducted using DLS analysis. The composition of the element is determined through EDAX analysis [109]. XRD is performed to recognize the size of the crystallite. FT-IR spectroscopy is used to detect the residues on the surface and the functional groups—such as flavonoids, phenols, and hydroxyls—which bond with the surface of the nanoparticles throughout the process of the synthesis for an effective reduction and stabilization. 

## 6. Antibacterial Activity of Flower-Derived NPs

NPs should come in contact with the bacterial cells to show the antibacterial function. The NPs pass through the membrane of the bacteria, add up along the pathway for metabolism, and influence the activity of a cell [110]. Subsequently, NPs associate with the elementary components of the bacterial cell, like DNA, lysosomes, ribosomes, and enzymes, and result in oxidative stress, heterogeneous altera tions, variations in the permeability of the cell membrane, disorders related to the balance of electrolytes, an inhibition of enzymes, a deactivation of proteins, and variations in the expression of the gene.

Cell walls and membranes are significant protective checkpoints for bacterial resistance to the outside environment, and the cell wall of the bacteria plays a vital function in sustaining the bacteria’s normal shape. The parts of the cell membrane of both Gram-positive and Gram-negative bacteria use diverse pathways for the adsorption of NPs [111]. Lipopolysaccharides (LPS) are an exclusive structure of the Gram-negative bacteria cell wall, which offers an area that is negatively charged for attracting NPs. On the other hand, the presence of teichoic acid is noted in Gram-positive bacteria cell walls; hence, NPs circulate throughout the phosphate molecular chain and avoid aggregation. NPs are more effective against Gram-positive than against Gram-negative bacteria, as their cell wall is made up of LPS, lipoproteins, and phospholipids, which produce a barrier that only permits the entry of macromolecules. On the other hand, cell membrane damage and cell death occur in Gram-positive bacteria, as its cell wall contains a thin sheet of peptidoglycan, teichoic acid, and ample pores, which permit the entry of foreign molecules [110].

The synthesis of AuNPs from *Plumeria alba* flower extract was conducted by adding 5 mL of flower extract to 45 mL of 0.002 M AuCl_4_ solution [104]. The process was continued for 3–4 h in the dark, until a pale-yellow solution was obtained. Synthesized AuNPs exhibited a higher antibacterial activity, performing a synergistic interaction with antibiotics—such as imipenem, vancomycin, and norfloxacin—against *Escherichia coli*. However, AuNPs synergistic to vancomycin and norfloxacin showed more antifungal activity against *Aspergillus flavus*. Iron nanoparticles were found to be efficient for the inhibition of bacterial growth, and the maximum zone of inhibition was observed for *E. coli* (21.8±0.2 mm), followed by *Staphylococcus aureus* (20.2 ± 0.3 mm) [87]. The *Catharanthus roseus* flower has been used for the synthesis of AgNPs and showed a potential antibacterial activity against *Bacillus subtilis*, *E. coli, Klebsiella pneumoniae, Pseudomonas putida*, and *S. aureus* [79]. Padalia et al. (2014) found that AgNPs formed from the flower extract of *Tagetes erecta* showed more antibacterial activity against *S. aureus* than against *Bacillus cereus* [95]. Lee et al. (2019) reported that *Tussilago farfara* flower bud extract, containing sesquiterpenoids, was efficiently utilized as a reducing agent for AgNPs synthesis [80]. The surface plasmon resonance peak of these silver NPs was observed at 416 nm on a UV–vis spectrophotometer, and TEM images revealed the shape of these nanoparticles as spherical, with a mean size of 13.57± 3.26 nm. These AgNPs displayed a better antibacterial activity in both Gram-negative and Gram-positive bacteria than the extract, and the maximum recorded antibacterial activity was against vancomycin-resistant enterococci (Van-A type), i.e., *Enterococcus faecium*. Sharma et al. (2016) synthesized spherical zinc oxide nanoparticles (ZnO NPs), with a size of 2–4 nm, from fallen *Jacaranda mimosifolia* flower aqueous extract (JMFs) [83]. In GC-MS analysis, the oleic acid in the flower extract was found to act as a reducing and capping agent, and the presence of oleic acid stabilized ZnO NPs, showing antibacterial activity against both Gram-positive *E. faecium* and Gram-negative *E. coli* bacteria. Abdallah et al. (2019) found that magnesium oxide (MgO) NPs formed from the flower extract of *Rosmarinus officinalis* L. showed a strong inhibitory effect against biofilms of a rice pathogen, *Xanthomonas oryzae* pv. *oryzae* strain GZ 0005 [86].

## 7. Antioxidant Potentials of Flower-Derived NPs

The presence of a variety of phytochemicals in flowers allows their extracts to contain antioxidant properties. A flower extract of *Cassia angustifolia* contains carbonyls, phenols, nitro compounds, alkane compounds, aromatics, alkyl halides, and many other aromatic phyto-compounds, which may act as reducing, capping, and stabilizing agents for AgNPs synthesis [92]. The DPPH potential of the synthesized AgNPs showed an IC_50_ value of 47.24μg/mL. On the other hand, theAgNPs H_2_O_2_-IC_50_ value was found to be 78.10μg/mL, while the FRAP-IC_50_ value was recorded to be 63.21μg/mL. The phyto-synthesized AgNPs induced 50% (IC_50_) of the anti-cancer activity against MCF 7 cells at a concentration of 73.82μg/mL, and *C. angustifolia* flower aqueous extract exhibited only a moderate activity against the tested cell line [92].

The AgNPs synthesized from flower extract of *Caesalpinia pulcherrima* were evaluated for antioxidant activity by ABTS cation radical scavenging activity, DPPH-free radical, FRAP, super oxide anion radical and reducing power assessment and showed that AgNPs were more effective in scavenging a variety of reactive oxygen species (ROS) [94]. However, at higher concentrations, the AgNPs resulted in decreased the cell viability of the HeLa cell line, and at a concentration of 200 µg/mL, AgNPs exhibited their maximum inhibition (18%), while at a concentration of 50 µg/mL, the cell viability was 23%. Whereas, an *in vivo* genotoxicity study showed that at a lower concentration, AgNPs do not cause any visibly harmful effects. 

## 8. Catalytic Properties of Flower-Derived NPs

Generally, 4-nitrophenol and its derivatives are used in the production of herbicides, insecticides, and synthetic dyestuffs, and they can badly harm the ecosystem as a general organic pollutant of wastewater [8]. As a result of its toxic and inhibitory nature, 4-nitrophenol is considered to have a huge risk to the environment. Therefore, the reduction of these pollutants must be crucial. The 4-nitrophenol reduction product (i.e., 4-aminophenol)has been used as a mediator for paracetamol, sulfur dyes, rubber antioxidants, the making of black/white film developers, corrosion inhibitors, and precursors in antipyretic and analgesic drugs [112,113]. The use of NaBH_4_ as a reductant and a metal catalyst for Au NPs, AgNPs, CuO NPs, and Pd NPs is the easiest and most effective approach to reduce 4-nitrophenol [114,115,116,117]. Methylene blue (MB), which is the member of the thiazine class of dyes, is another heterocyclic aromatic industrial pollutant [118]. The ingestion of MB in human body has been reported to restrict oxidase enzymes in the body, which may lead to grave disorders, i.e., toxicity of the central nervous system, gastrointestinal infections and decolorization of the brain parenchyma [119,120]. During the reduction of MB, NaBH_4_ acts as a reducing agent, and NPs act as an absorbent [121].

Nayan et al. (2018) synthesized AuNPs using *Mangifera indica* flower extract (MIFE) [82]. In the aqueous phase, these AuNPs showed a high nano-catalysis to reduce 4-nitrophenol to 4-aminophenol through the use of NaBH_4_ at room temperature. *Ipomoea digitata* (ID) flower extract-synthesized AgNPs were studied to check their catalytic activity against MB dye, with NaBH_4_ as a model reducing agent [96]. The oxidized state of MB (blue color) becomes colorless when reduced to leuco-methylene blue (LMB). The addition of ID-AgNPs to the reaction mixture resulted in the formation of an intermediate between MB dye and BH_4_ ions. This study showed a good catalytic ability of synthesized nanoparticles, as the catalytic reduction of MB dye by NaBH_4_ was completed within 15 min, indicating a prospective application of the ID-AgNPsfor environmental remediation.

## 9. Insecticidal Properties of Flower-Derived NPs against Parasites

The insecticidal potential of flower-derived nanoparticles has also been identified. Cadmium nanoparticles (CdNPs) (10 ppm) synthesized from marigold petal extract showed a mortality rate of 68.9% against *Aedes albopictus*, while at the same concentration, CdNPs showed a 100% mortality rate after 72 h of treatment against *A. albopictus* [84]. Similarly, another study reported the killing potential of *Chryasanthemum indicum* L. floral extract-derived AgNPs [97]. In this study, different concentrations of *C. indicum* aqueous extract and synthesized AgNPs were tested against *Anopheles stephensi* mosquito larvae and pupae, and the maximum mortality was observed with the synthesized AgNPs against the vector, *A. stephensi* (LC_50_ = 5.07, 10.35, 14.19, 22.81, and 35.05 ppm; LC_90_ = 29.18, 47.15, 65.53, 87.96, and 115.05 ppm). *Calotropis gigantean* flower extract-derived TiO_2_ NPs showed LC_50_ values of 9.15 mg/L and 5.43 mg/L against the larvae of *Haemaphysalis bispinosa* and against *Rhipicephalus microplus*, respectively [85]. 

## 10. Challenges in the Use of Flower-Mediated Nanoparticles 

Technical barriers are the obstructions that are involved during the synthesis of flower-mediated nanoparticles. While green nanoscience has gained significant attention, efforts are still being made to standardized the protocols for the synthesis of uniform nanoparticles. Further advancements involving the use of tools and techniques for the scaled-up production of NPs through green synthesis need to be identified to design commercially feasible production technology at the industrial scale. Another pivotal issue regarding the large-scale use of green synthesized nanoparticles is nano-toxicity, which has to be addressed stringently. The toxicology and analysis protocols have to be developed and updated constantly to reflect the need of the application. Furthermore, the uncertainty and ambiguity associated with the regulatory bodies and laws has to be clearly understood to allow for the use and commercialization of ecologically safe nano-based products. The end market demands need to be made clear, as there are only limited numbers of commercial grade products that can be compared to conventional materials in terms of performance [122].

A unique idea, which still needs to be developed and established, is the use of flowers in the green synthesis of nanoparticles, as this research is still restricted to the synthesis of Au and Ag NPs. To further strengthen this field, it is important to create monodispersed nanoparticles—such as CdS, ZnO, TiO_2_, and Fe_3_O_2_. More studies are required to recognize the various components that may lead to the reduction of metal ions. In the literature, it has been reported that proteins are responsible for the equilibrium, but it is very difficult to recognize the proteins responsible for the functionalization of these nanoparticles [123].

## 11. Conclusions

The use of biological materials for the production of nanoparticles has a great potential as a cost-effective and eco-friendly synthesis method for novel and innovative nanomaterials. Non-hazardous biological wastes also play a crucial role in green synthetic protocols for the generation of nanoparticles [36]. The green chemistry approach is completely different from the conventional physical and chemical processes, which frequently utilize environmentally corrosive agents with the ability to cause cytotoxicity, environmental toxicity, and carcinogenicity. On the other hand, the flower-mediated green synthesis of NPs is a vigorous method that does not require any specific isolation and maintenance procedures, which are needed in bacteria-, fungi-, or algae-based nanoparticle synthesis approaches. Flower-induced nanoparticles can exhibit specialized properties, including antimicrobial, antioxidant, catalytic, and cytotoxic activities.The present study intends to highlight the potential of flower-derived metallic nanoparticles. Of all the studied nanoparticles, Au and AgNPs were shown to be the best potential nanoparticles in terms of their effective antibacterial, antioxidant, and insecticidal activities. Bio-accumulation and toxicity are the two challenges associated with green metallic nanoparticles that prevent their use as therapeutic agents in humans and that need to be resolved through scientific intervention. With further improvement, the flower-mediated green synthesis of nanoparticles may offer important, ecofriendly end products, with wide applications, as compared to the harsh and lethal procedures used at present for the synthesis of nanoparticles.

## Figures and Tables

**Table 1:** Different types of reducing and stabilizing agents used in the flower-mediated biosynthesis of nanoparticles.

Nanoparticle Types	Reducing Agent	Stabilizing Agent	Specific Temperature	Ref
Silver	chlorine ions	proteins present in the flower	RT	[75,76]
	water-soluble heterocyclic components, polyols, and certain proteins	flower	RT	[77]
	flower	flower	40 °C	[78]
	flower	flower	60 °C	[79]
	sesquiterpenoids	DMEM + FBS	80 °C	[80]
Gold	sesquiterpenoids	DMEM + FBS	80 °C	[80]
	flower	flower	40 °C	[81]
	polyphenols and flavonols	flower	25–60 °C	[82]
Zinc	flower	flower	microwave irradiation	[83]
Cadmium	tannins, flavonoids, alkaloids, and carotenoids	flower	RT	[84]
Titanium	flower	flower	60 °C	[85]
Magnesium	flower	flower	70 °C	[86]
Iron	flower	flower	RT	[87]

RT—room temperature; DMEM—Dulbecco’s modified eagle medium; FBS—fetal bovine serum.

**Table 2:** Applications of silver NPs synthesized from various flower varieties.

Family	Flower Variety	Applications	Ref
Fabaceae	*Lablab purpureus*	Antibacterial activityagainst *Escherichia coli* and *Staphylococcus aureus*	[90]
Apocynaceae	*Plumeria rubra*	Antibacterial activity against *Escherichia coli* and *Bacillus sp.*	[91]
Apocynaceae	*Catharanthus roseus*	Antibacterial activity against *Escherichia coli, Pseudomonas putida, Staphylococcus aureus, Klebsiella pneumoniae,* and *Bacillus subtilus*	[79]
Fabaceae	*Cassia angustifolia*	Antioxidant and cytotoxicity activity	[92]
Apocynaceae	*Allamanda cathartica*	Antioxidant activity and antibacterial activity against *Salmonella typhimurium, Staphylococcus aureus, Escherichia coli*,and *Klebsiella pneumoniae*	[93]
Malvaceae	*Malva sylvestris*	Antibacterial activity against *Escherichia coli*, *Staphylococcus aureus*, and *Streptococcus pyogenes*	[76]
Fabaceae	*Caesalpinia pulcherrima*	Antibacterial activity against *Staphylococcus aureus*; antifungal activity against *Candida glabrata*; antioxidant activity; cytotoxicity activity	[94]
Asteraceae	*Tussilago farfara*	Antibacterial activity against *Enterococcus faecium*; cyrotoxicity activity	[80]
Asteraceae	*Tagetes erecta*	Antibacterial activity against *Escherichia coli* and *Pseudomonas aeruginosa*; antifungal activity against *Candida albicans*	[95]
Sapotaceae	*Madhuca longifolia*	Antibacterial activity against *Bacillus cereus* and *Staphylococcus saprophyticus*	[78]
Malvaceae	*Hibiscus rosa-sinensis*	antibacterial activity against *Aeromonas hydrophila*	[77]
Convolvulaceae	*Ipomoea digitata* Linn	Antibacterial activity against *Staphylococcus epidermidis*; catalytic activity against methylene blue	[96]
Asteraceae	*Chrysanthemum indicum* L.	Larvicidal and pupicidalactivity against *Anopheles stephenis*	[97]

**Table 3:** Applications of gold NPs synthesized from various flower varieties.

Family	Flower Variety	Applications	Ref
Apocynaceae	*Plumeria alba* Linn	Antibacterial activity against *Escherichia coli*	[104]
Thymelaeaceae	*Gnidia glauca*	Chemocatalytic activity against 4-nitrophenol	[81]
Anacardiaceae	*Mangifera indica*	Catalytic activity against 4-nitrophenol	[82]
Asteraceae	*Tussilago farfara*	Antibacterial activity against *Enterococcus faecium*; cyrotoxicity activity	[80]

**Table 4:** Applications of other types of NPs synthesized from various flower varieties.

Family	Flower Variety	Types of Nanoparticles Synthesized	Applications	Ref
Sapotaceae	*Mimusops elengi*	Copper	Antibactrial activity against *Escherichia coli, Streptococcus, Staphylococcus*, *Pseudomonas,* and *Bacillus subtilis*; antifungal activity *Aspergillus flavus, Candida albicans, Penicillium* and *Aspergillus fumigates*; antioxidant activity; thrombolytic activity; anti-larval activity; cytotoxicity activity; heavy metals removal	[106]
Fabaceae	*Piliostigma thonningii*	Iron	Antibacterial activity against *Escherichia coli* and *Staphylococcus aureus*	[87]
Oleaceae	*Nyctanthes arbor-tristis*	Zinc	Antifungal activity against *Alternaria alternate, Aspergillus niger, Botrytis cinerea, Fusarium oxysporum,* and *Penicillium expansum*	[107]
Myrtaceae	*Syzygium aromaticum*	Zinc	Antifungal activity against *Fusarium graminearum*	[105]
Bignoniaceae	*Jacaranda mimosifolia*	Zinc	Antibacterial activity against *Enterococcus faecium*	[83]
Asteraceae	*Tagetes sp.*	Cadmium	Larvicidal activity against *Aedes albopictus*	[84]
Apocynaceae	*Calotropis gigantean*	Titanium	Acaricidal activity against *Rhipicephalus microplus* and *Haemaphysalis bispinosa*	[85]
Lamiaceae	*Rosmarinus officinalis* L.	Magnesium	Antibacterial activity against *Xanthomonas oryzae* pv. *oryzae*	[86]

**Table 5:** Synthesis and characterization of metallic NPs from various flower varieties.

Family	Flower Variety	Types of Nanoparticles Synthesized	Methods Used for NPs Characterization											Size	Morphology	Ref
			UV–vis	TEM	SEM	FT-IR	XRD	EDX	DLS	Zeta Potential	HRTEM	AFM	GC-MS			
Fabaceae	*Lablab purpureus*	Silver	√	-	√	√	√	-	-	-	-	-	-	5–50 nm	Spherical	[90]
Sapotaceae	*Mimusopselengi*	Copper	√	-	√	√	√	-	-	-	-	-	-	42–90 nm	Rod and spherical	[106]
Fabaceae	*Piliostigma thonningii*	Iron	√	-	√	√	√	-	-	-	-	-	-	20–100µm	Rod and spherical	[87]
Oleaceae	*Nyctanthes arbor-tristis*	Zinc	√	√	-	√	√	-	√	-	-	-	-	12–32 nm	Aggregate	[107]
Apocynaceae	*Plumeria rubra*	Silver	√	√	-	-	-	-	-	-	-	-	-	20–80 nm	Spherical and irregular	[91]
Apocynaceae	*Catharanthus roseus*	Silver	√	√	-	√	-	-	-	-	-	-	-	6–25 nm	spherical	[79]
Fabaceae	*Cassia angustifolia*	Silver	√	-	√	√	√	√	-	-	-	-	-	10–80 nm	Spherical	[92]
Apocynaceae	*Plumeria alba* Linn	Gold	√	-	-	-	-	-	-	-	√	-	-	20–30 and 80–150 nm	Spherical	[104]
Myrtaceae	*Syzygium aromaticum*	Zinc	√	√	√	√	√	-	-	-	-	-	-	30–40 nm	Triangular and hexagonal	[105]
Thymelaeaceae	*Gnidia glauca*	Gold	√	√	-	√	√	-	√	-	√	-	-	50–150 nm	Spherical	[81]
Apocynaceae	*Allamanda cathartica*	Sliver	√	√	-	√	√	-	-	-	-	-	-	39 nm	Spherical	[93]
Malvaceae	*Malva sylvestris*	Silver	√	√	-	√	-	√	-	-	-	√	-	20–40 nm	Spherical	[76]
Fabaceae	*Caesalpinia pulcherrima*	Silver	√	√	-	√	√	-	-	-	-	-	-	12 nm	Spherical	[94]
Asteraceae	*Tussilago farfara*	Silver and Gold	√	√	-	-	√	-	-	√	-	√	-	13.57 and 18.20 nm	Spherical	[80]
Anacardiaceae	*Mangifera indica*	Gold	√	√	-	-	√	√	-	-	√	-	-	10–60 nm	Spherical	[82]
Asteraceae	*Tagetes erecta*	Silver	√	√	-	√	√	-	-	-	-	-	-	10–90 nm	Spherical, hexagonal, and irregular	[95]
Sapotaceae	*Madhuca longifolia*	Silver	√	√	√	√	√	√	-	√	-	-	-	30–50 nm	Spherical and oval	[78]
Bignoniaceae	*Jacaranda mimosifolia*	Zinc	√	-	-	√	√	-	-	-	√	-	√	2–4 nm	Spherical	[83]
Malvaceae	*Hibiscus rosa-sinensis*	Silver	√	-	√	√	-	-	-	-	-	-	-	5–40 nm	Spherical	[77]
Convolvulaceae	*Ipomoea digitata* Linn	Silver	√	-	√	√	√	√	-	-	-	-	-	111 nm	Spherical	[96]
Asteraceae	*Tagetes sp.*	Cadmium	√	-	√	√	-	-	-	-	-	-	-	50 µm	Spherical	[84]
Apocynaceae	*Calotropis gigantean*	Titanium	-	-	√	√	√	√	-	-	-	-	-	160–220 nm	Spherical	[85]
Lamiaceae	*Rosmarinus officinalis* L.	Magnesium	√	√	-	√	√	-	-	-	-	-	-	20 nm	Spherical	[86]
Asteraceae	*Chrysanthemum indicum* L.	Silver	√	√	-	-	√	√	-	-	-	-		25–59 nm	Spherical	[97]

UV–vis–Ultraviolet-visible spectroscopy; TEM–Transmission electron microscopy; SEM–Scanning electron microscopy; FT-IR–Fourier-transform infrared spectroscopy; XRD–X-ray powder diffraction; EDX–Energy dispersive X-ray spectroscopy; DLS–Dynamic light scattering; HRTEM–High-resolution transmission electron microcopy; AFM–Atomic force microscopy; GC-MS–Gas chromatography-mass spectroscopy.
